# Living in rural New England amplifies the risk of depression in patients with HIV

**DOI:** 10.1186/1471-2334-9-25

**Published:** 2009-03-05

**Authors:** Siddharth H Sheth, Paul T Jensen, Timothy Lahey

**Affiliations:** 1 The Center for Education, The Dartmouth Institute for Health Policy and Clinical Practice, Lebanon, NH, USA; 2 Dartmouth Medical School, Hanover, NH, USA; 3 Department of Microbiology and Immunology, Dartmouth Medical School, Hanover, NH, USA; 4 Department of Medicine, Dartmouth-Hitchcock Medical Center, Lebanon, NH, USA

## Abstract

**Background:**

The importance of depression as a complication of HIV infection is increasingly understood, and people living in rural areas are at increased risk for depression. However, it is not known whether living in rural areas amplifies the risk of depression in patients with HIV.

**Methods:**

We compared the prevalence of depression between rural and metropolitan HIV patients seen at the Dartmouth-Hitchcock HIV Program in a retrospective cohort study. Using the validated Rural-Urban Commuting Area Score, we categorized patients as living in small town/rural areas, micropolitan or metropolitan towns. Then, using a multivariate logistic regression model to adjust for demographic factors that differed between rural and metropolitan patients, we estimated the impact of living in rural areas on the odds of depression.

**Results:**

Among 646 patients with HIV (185 small town/rural, 145 micropolitan, 316 metropolitan), rural patients were older, white, male, and men who have sex with men (ANOVA, F-statistic < 0.05). The prevalence of depression was highest in rural patients (59.5 vs. 51.7 vs. 41.2%, F statistic < 0.001), particularly rural patients on antiretroviral therapy (72.4 vs. 53.5 vs. 38.2%, F-statistic < 0.001. A multivariate logistic regression model showed that the odds of depression in rural patients with HIV were 1.34 (P < 0.001).

**Conclusion:**

HIV-infected patients living in rural areas, particularly those on antiretroviral therapy, are highly vulnerable to depression.

## Background

Since its identification in 1981 [1], HIV infection in the United States has spread from coastal cities to inland rural towns [2-4]. HIV care in rural areas is inferior to HIV care in urban areas: rural patients cite greater obstacles to care, see less experienced HIV providers, and are less likely to receive antiretroviral treatment or prophylaxis against *Pneumocystis jiroveci *pneumonia (PCP) than their urban counterparts [5,6]. Accordingly, we recently found that rural patients with HIV have higher mortality rates [7].

Depression is the most common neuropsychiatric complication of HIV disease [8]. The point prevalence of major depression in HIV infected individuals is 4–10%, with lifetime prevalence estimated between 22–45% [8]. HIV-infected patients with depression experience poorer physical and social well-being and greater bodily pain. Lastly, HIV patients with depression are less adherent to antiretroviral therapy (ART), and have lower CD4 counts along with an unfavorable general prognosis [9-14].

Given the increasing prevalence of HIV in rural areas, and prior data suggesting that rural patients without HIV infection are at high risk of depression [15], we examined whether living in rural areas amplifies the risk of depression in HIV-infected patients from New England.

## Methods

### Subjects and data acquisition

We conducted a retrospective study of 646 adult HIV patients seen from 1995–2005 at four federally funded clinics encompassing the Dartmouth-Hitchcock HIV Program. Regardless of their location of residence, patients received care from the same multidisciplinary HIV team consisting of physicians, nurses, psychologists, nutritionists and other support staff. We included all patients in our HIV program in this analysis who were seen in our clinics more than one time throughout our study period, with the exception of those with inadequate residency records. Demographic and clinical characteristics were assessed using a retrospective medical records review following approval by the Committee for Protection of Human Subjects at Dartmouth College and in accordance with the Declaration of Helsinki. We used de-identified data and therefore were exempted from obtaining informed consent from our study subjects.

### Study endpoints

We assessed the prevalence of the diagnosis of depression by a physician at any time point according to the medical records. We did not require that our study population meet specific diagnostic criteria for depression. To characterize patients' residency, we utilized the Rural-Urban Commuting Area (RUCA) score, a validated categorical measure based on population and commuting patterns [16]. According to standard RUCA guidelines, we used three categories: small town/rural areas (population < 10,000), micropolitan (population 10,000–49,999), and metropolitan (population > 50,000). In subset analyses, we combined the two non-metropolitan RUCA categorizations, designating subjects in this combined group as 'rural.'

### Additional variables

To assess the impact of other clinical variables on the relationship between rurality and depression, we also abstracted the following from the medical record: age in 2007, race, sex, CD4 counts, ART history, and measures for driving time and distance. We categorized subjects based on CD4 count at their first and last data point according to two thresholds: below 200 cells/mm^3^, in which patients were designated as having AIDS, and below 350 cells/mm^3^, which signified eligibility to start ART during the evaluation period. Patients were classified as having AIDS if, at any time in their medical record, they met the 1993 CDC AIDS Surveillance case definitions of AIDS [17]. We also recorded whether they were ever diagnosed with an opportunistic infection during the eleven year study period. Lastly, we used Mapquest.com to determine the distance and time between the zip codes of the patient's home location and their preferred clinic location.

### Statistical Analysis

When comparing depression between the three categories of the RUCA score, we used a one-way analysis of variance (ANOVA) model. When conducting univariate comparisons between two groups, we used a *t *test. Variables that were significantly different between groups in univariate analyses were incorporated a priori into a multivariate logistic regression analysis. The likelihood ratio test was used to detect interaction between the terms used in multivariate analyses. For all analyses, we considered P values < 0.05, and ANOVA F-statistic values < 0.05, to be statistically significant. All statistical analyses were performed using STATA 9.0 (College Station, TX).

## Results

### Study Population

Table 1 highlights demographic characteristics and HIV information of patients in the Dartmouth-Hitchcock HIV Program from 1995–2005 according to RUCA category of residency. Patients living in rural areas were older and more likely to be male, white, and men who have sex with men.

**Table 1:** Characteristics of patients in Dartmouth-Hitchcock HIV Program by RUCA categorization (1995–2005)

	Small Town/Rural (n = 185)	Micropolitan (n = 145)	Metropolitan (n = 316)	F Statistic by ANOVA
**Age, years**	43.6	43.0	41.5	0.019

**Male, %**	79.6	80.3	64.4	< 0.001

**Population of town, mean**	5,598	14,389	79,168	< 0.001

**Race, %**				

White	94.1	92.4	77.5	< 0.001

Black	5.41	5.52	21.5	< 0.001

Asian	0.0	1.38	0.95	0.324

**Risk Factor, %**				

Heterosexual	24.1	24.8	36.8	0.007

Intravenous drug use	16.5	19.0	17.5	0.857

Men who have sex with men	58.2	60.3	46.4	0.011

Hemophilia	3.2	0.8	0.8	0.113

**HIV disease status**				

CD4 T cell count at diagnosis, cells/mm^3^	378.0	321.6	373.9	0.178

CD4 T cell count at last follow-up, cells/mm^3^	455.3	426.4	444.0	0.672

HAART, %	47.0	43.0	40.8	0.533

Opportunistic infections, %	34.1	38.9	22.9	< 0.001

Acquired immunodeficiency syndrome, %	61.6	64.6	50.8	0.007

**Follow-Up Time, years**	10.5	9.30	8.30	< 0.001

**Access to Care**				

MapQuest Travel Time, minutes	60.7	51.7	33.5	< 0.001

MapQuest Travel Distance, miles	47.2	39.9	25.6	< 0.001

### Depression in rural patients with HIV: prevalence and risk factors

**Figure 1:**
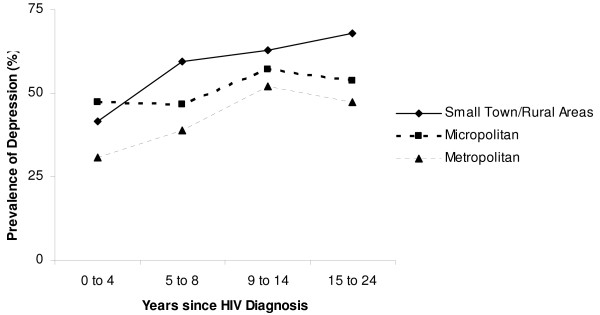
**Prevalence of depression based on time since HIV diagnosis based on patient residence**. Prevalence of depression based on time since HIV diagnosis in patients living in small town/rural areas, micropolitan, and metropolitan areas.

We found that the prevalence of depression was highest in patients living in small town/rural areas, and in micropolitan towns, compared to metropolitan areas, respectively (59.5 vs. 51.7 vs. 41.0%, F statistic < 0.001). The major risk factors for depression were white race and intravenous drug use (Table 2). Figure 1 indicates that the prevalence of depression increased over time in all patients; this trend was most pronounced in patients living in small town/rural areas.

**Table 2:** Depression risk factors for all individuals in Dartmouth-Hitchcock HIV Program (1995–2005)

	Depression (n = 314)	No Depression (n = 331)	P value by t test
**Age, years**	42.9	42.0	0.189

**Male, %**	74.4	70.6	0.312

**Race, %**			

White	90.1	81.2	0.001

Black	9.2	17.2	0.003

Asian	0.3	1.2	0.198

**HIV risk factor, %**			

Heterosexual	24.3	36.6	0.002

Intravenous drug use	21.7	13.2	0.009

Men who have sex with men	56.9	49.1	0.068

Hemophilia	0.4	2.7	0.028

**HIV disease status**			

Acquired immunodeficiency syndrome, %	54.1	48.0	0.121

Opportunistic infection, %	29.5	29.8	0.934

CD4 T cell count at last follow-up, cells/mm^3^	433.4	453.5	0.384

**Follow-Up Time, years**	10.2	8.12	< 0.001

**Access to Care**			

MapQuest Travel Time, minutes	43.3	47.3	0.206

MapQuest Travel Distance, miles	33.8	36.2	0.358

### Modifiers of Depression Prevalence

We investigated whether the prevalence of depression varied as a function of HIV disease progression. Most recent CD4 counts were similar among patients with and without depression (433.4 vs. 453.5 cells/mm^3^, P = 0.384). Similarly, CD4 counts were similar in subjects from small town/rural areas, micropolitan and metropolitan towns (455.3 vs. 426.4 vs. 444.0 cells/mm^3^, F-statistic 0.672). Further, we found that the relationship between depression and rural residency persisted when stratified by CD4 count strata (Table 3). When we grouped patients from small town/rural areas and micropolitan towns together and compared them to metropolitan patients, the prevalence of depression was greater in rural patients (CD4 counts < 200 cells/mm^3^, 55.0 vs. 40.0%, P = 0.073; CD4 counts 200–350 cells/mm^3^, 60.9 vs. 34.8%, P = 0.002; and, CD4 counts > 350 cells/mm^3^, 54.4 vs. 43.2%, P = 0.033).

**Table 3:** Prevalence of Depression in Rural and Metropolitan Areas Stratified by CD4 Cell Count and Use of Antiretroviral Therapy

	Small Town/Rural Areas	Micropolitan	Metropolitan	ANOVA F-Statistic
**CD4 count, cells/mm^3^**				

< 200	53.3	57.1	40.0	0.191

200–350	74.4	43.3	34.8	< 0.001

> 350	55.9	52.5	43.2	0.033

**ART Use**				

Yes	72.4	53.5	38.2	< 0.001

No	60.0	50.9	46.9	0.211

We next explored how ART impacts the relationship between rural residency and depression. Here, we found that the prevalence of depression was greater in patients on ART compared to those not on ART (54.6 vs. 39.8%, P = 0.014). When stratifying our analysis of the relationship between depression and living in rural areas by antiretroviral treatment status, we found that the relationship was significant only in rural patients on ART (Table 3). Similarly, when grouping patients from small town/rural areas and micropolitan towns together, we found that the prevalence of depression was higher in rural patients on ART (64.8 vs. 38.3%, P < 0.001) but not in rural patients off ART (55.9 vs. 46.8%, P = 0.150).

Next, we explored if the prevalence of depression differed between the four Dartmouth-Hitchcock HIV clinic sites. Table 4 shows that depression was a more common diagnosis in our rural clinics (ANOVA F statistic < 0.001).

**Table 4:** Diagnosis of Depression by Clinic Location

Clinic Location	City Population	RUCA Categorization	Patients with Depression, n (%)
Manchester, NH	107,006	Metropolitan	99/219 (45.2)

Lebanon, NH	12,688	Micropolitan	164/322 (50.9)

Brattleboro, VT	12,006	Micropolitan	36/45 (80.0)

Nashua, NH	86,605	Metropolitan	15/58 (25.9)

### Multivariate Models

In a logistic regression model comparing the prevalence of depression in small town/rural areas, micropolitan, and metropolitan patients, the unadjusted odds of depression in rural patients was 1.46 (CI 1.22–1.75, P < 0.001), indicating increasing risk in increasingly rural areas. Adjusting for age, sex and HIV acquisition risk factor, we found that the odds of depression in rural patients was 1.34 (CI 1.09–1.65, P < 0.001). Table 5 shows these and additional logistic regression models, all of which show a significant relationship between living in rural areas and depression.

**Table 5:** Regression Analyses: Effects of Rural Residence on the Likelihood of Depression in Patients with HIV

Factors Controlled	Odds Ratio (OR)	Confidence Interval (CI)	P-value
1. Unadjusted	1.46	1.22–1.75	< 0.001

2. Age, Male, White, MSM	1.34	1.09–1.65	< 0.001

3. Same as #2 + Clinic Location	1.36	1.10–1.68	0.005

4. Same as #2 + Follow-up Time	1.30	1.05–1.61	0.015

## Discussion

In the United States today, rural patients with HIV face greater obstacles to medical care, receive less expert HIV care, experience reduced access to antiretroviral treatment, and have higher mortality rates [3,18,19]. We now show that the prevalence of depression is higher in HIV patients living in rural areas, particularly those on ART. In fact, we observed a gradient of increasing depression moving from metropolitan to micropolitan towns and into small town/rural areas.

The prevalence of depression we observed in rural patients with HIV is higher than rates reported in other populations. Previous studies have cited a prevalence of depression of 5.2% in the general population [20], 6.1% in rural populations [15], and a 9.4% in HIV-infected individuals [20]. While the depression rates in HIV-infected patients in our study cannot be compared directly to these figures, the 59.5% prevalence of depression in HIV patients from small town/rural areas over an 11-year period clearly suggests that these patients are highly vulnerable to this devastating disease.

Further, we found that the prevalence of depression was highest in rural patients on ART. While this finding may recapitulate the prior observation that depression is more common with advanced HIV [21,22], it is also possible that the greater vulnerability to depression of rural patients on ART stems from treatment fatigue. Our finding that depression prevalence remains elevated for rural HIV patients across multiple CD4 strata supports this possibility.

HIV-infected rural patients are demographically distinct from HIV patients living in metropolitan areas. Adjusting for these factors in the multivariate model did not impact the clear relationship between living in rural areas and depression. This suggests that demographics alone do not explain the higher depression prevalence in rural HIV patients in New England. Additionally, it newly defines risk factors for depression in this high risk cohort.

We were unable to determine why the prevalence of depression is higher in rural HIV patients. However, we hypothesize that inadequate social support is a major contributor. Two findings support this hypothesis: inadequate social support is linked to higher risk of depression in patients without HIV [23,24], and there are fewer social support resources for HIV care in rural areas [6,25]. Since patients with HIV are vulnerable to unstable employment [26], one source for poor social support is limited access to insurance benefits and associated community resources. Furthermore, our clinical experience suggests that support services for HIV care are weak, especially in the small town/rural areas where the prevalence of depression in our patients was the highest. Similarly, because poor social support is known to predict depression in patients on ART [27], we suspect that inadequate local social support for HIV care may contribute to the extremely high prevalence of depression in rural HIV patients on ART. This is a critical point, because depression undermines ART adherence and is correlated with poorer HIV prognosis [28-30]. Stigma is another major likely contributor to the increasing prevalence of depression in rural areas. Others have shown that rural patients are more likely to encounter AIDS-related stigma [26], and we posit this may contribute to the great vulnerability to depression we observed in our rural patients.

Our study has important methodological limitations that should be acknowledged. First, the diagnosis of depression in patients in this study was not based on formal criteria; rather, we relied on clinician diagnosis. Thus, provider variability in diagnosis may impact the definition of depression in our study population. However, using this operational definition of depression reflects real world clinical practice [31], and as such is likely to capture a real burden of psychiatric disease in our study population.

Second, we measured the prevalence of the diagnosis of depression over an eleven year period, not the point prevalence of depression. Therefore, our data are not amenable to survival analysis, and thus bias introduced by differential follow-up between groups is possible. One potential source for differential follow-up is migration. It is conceivable that patients from metropolitan areas left our cohort at a different rate compared to those living in rural areas; however, adjusting for follow-up time in our multivariate model did not undermine the significant relationship between living in rural areas and the prevalence of depression.

Furthermore, we did not characterize the severity of depression or use a rigorous validated screening tool. Thus, our data cannot capture the dynamic incidence or variable severity of the disease, and how this might vary geographically. Lastly, it is possible that individuals in our cohort acquired the diagnosis of depression prior to their HIV diagnosis, and as a result, a substantial proportion of the increased depression prevalence is independent of HIV disease. Nevertheless, it is clinically important that the prevalence of depression in rural HIV patients is much higher than that reported in either rural or HIV populations studied independently.

Importantly, we identified multiple risk factors for depression in HIV patients, including white race and intravenous drug use. However, when controlling for these risk factors in our multivariate analyses, the strong relationship between living in rural areas and the prevalence of depression remained. Therefore, we believe these results are generalizable despite the demographic differences between our rural HIV cohort and those seen in other rural areas of the United States [3,18,32].

We recognize that rurality is a necessarily subjective designation, but believe that the use of validated RUCA scale lends credence to our findings [16]. Additionally, the fact that we demonstrated a gradient of depression moving from metropolitan cities to micropolitan cities and to small town/rural areas further supports the idea that living in rural areas is an important risk factor for depression in patients with HIV.

## Conclusion

Rural patients with HIV are highly vulnerable to major depression, especially those from small town/rural areas. This risk is most pronounced in rural HIV patients on ART. We believe that poor social supports, HIV-related stigma, and antiretroviral treatment fatigue all contribute to the pronounced vulnerability of rural HIV patients to depression.

## Competing interests

The authors declare that they have no competing interests.

## Authors' contributions

SS performed the statistical analysis, drafted and edited the manuscript, and has given final approval for the manuscript to be published. PJ performed medical chart reviews for data acquisition, revised the manuscript critically, and has given final approval for the manuscript to be published. TL conceived the study, participated in the design and coordination of the study, drafted and edited the manuscript, and has given final approval for the manuscript to be published.

## Funding Sources

None

## Pre-publication history

The pre-publication history for this paper can be accessed here:

http://www.biomedcentral.com/1471-2334/9/25/prepub
